# A Mobile Device System for Early Warning of ECG Anomalies

**DOI:** 10.3390/s140611031

**Published:** 2014-06-20

**Authors:** Adam Szczepański, Khalid Saeed

**Affiliations:** AGH University of Science and Technology, Faculty of Physics and Applied Computer Science, 30 Mickiewicza Av., PL-30059, Krakow 004812, Poland; E-Mail: Adam.Szczepanski@fis.agh.edu.pl

**Keywords:** ambient intelligence, ECG anomalies, heart anomaly alert, ECG monitoring for mobile devices

## Abstract

With the rapid increase in computational power of mobile devices the amount of ambient intelligence-based smart environment systems has increased greatly in recent years. A proposition of such a solution is described in this paper, namely real time monitoring of an electrocardiogram (ECG) signal during everyday activities for identification of life threatening situations. The paper, being both research and review, describes previous work of the authors, current state of the art in the context of the authors' work and the proposed aforementioned system. Although parts of the solution were described in earlier publications of the authors, the whole concept is presented completely for the first time along with the prototype implementation on mobile device—a Windows 8 tablet with Modern UI. The system has three main purposes. The first goal is the detection of sudden rapid cardiac malfunctions and informing the people in the patient's surroundings, family and friends and the nearest emergency station about the deteriorating health of the monitored person. The second goal is a monitoring of ECG signals under non-clinical conditions to detect anomalies that are typically not found during diagnostic tests. The third goal is to register and analyze repeatable, long-term disturbances in the regular signal and finding their patterns.

## 1. Introduction

A little more than decade ago smartphones and tablets were only considered expensive gadgets. Nowadays, with the rapid development of the technology for both devices, many people cannot imagine their life without at least one of them. Currently they serve mostly for entertainment and socializing, although with the rapidly increasing computational power new possibilities for complex applications are opening for these devices. One such application, proposed in this paper, is the electrocardiogram (ECG) signal analysis during everyday activities.

The main purpose of the authors' current and future work is a creation of flexible and robust solution for monitoring ECG signal in real time for patients who are using mobile devices. The three main goals of such system are defined as follows:

- pattern matching analysis of ECG signal in real time for detection of rapid life threatening changes,
- finding anomalies which are usually not found during regular diagnostic procedures,
- registering and analyzing repeatable, long-term disturbances in the regular signal, finding their patterns and sending them to a doctor who is looking after the patient for manual verification of the diagnosis.

This work concentrates mainly on the first of the goals, describing a prototype of the authors' solution for faulty beat detection and demonstrating its possibility to be transferred onto mobile platforms. The second of the described goals was already implemented by authors [1]. The third goal can be achieved by further extending the software already created by the authors.

The paper consists of five main parts. In the first part the planned system is described in the context of the current state of the art. The possible problems that may occur during the creation of such a system are discussed. In the second part the authors describe their previous and current work, concentrating on the solutions that will be used in the proposed system, mainly the ECG signal analysis algorithms. The third part also focuses on working out ECG analysis software for mobile devices. The fourth part concentrates on tests performed using both simulation software and mobile implementation. In the last part the results, the planned solutions for the unsolved problems and the plans for near future work are discussed.

## 2. Background

To learn the basics about ECG signals, their analysis techniques, interpretation, typical placement of electrodes for measurement and most common heart diseases, the authors recommend using basic medical handbook tutorials, for example [2], which are designed for fast knowledge recalling during emergency conditions and thus are written in very clear, easily understandable and simplistic style.

**Figure 1.**
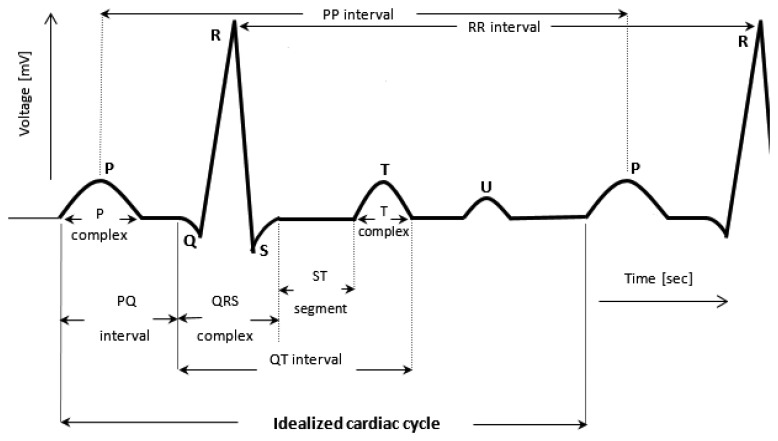
A part of an ECG waveform showing the cardiac cycle and its constituent patterns (illustrated on the basis of ECG signal description in [5,6]).

The schematic waveform of a single heartbeat in an ECG is presented in Figure 1. Points represented on the signal as P, Q, R, S and T are the main reference points in interpretation of ECG signal ([2,3]). The U Wave, which may appear in some people's heartbeats just after T Wave, may be skipped in further analysis because it does not include important information in the context of detection of rapid cardiac disturbances [4].

The analysis of the ECG is conducted according to the order described below [6].

In its normal work, the heart rhythm is of sinus type characterized among other features by the presence of P-complex before every QRS complex in a cycle and simultaneously the presence of QRS after every P complex. The heart impulse rate is characterized by heart agitation in average in the frequency of 60–100 beats per minute (bpm) for adults, although it might be normal, especially for athletes, to have resting bpm as low as 30. Deviations of the rate beyond this range can be treated as abnormal case. However, the limit frequencies should be taken individually. For the time duration of PR, QRS and QT parts in the cardiac cycle, a reasonable rule is to consider that the interval QT is less than a half of the distance between two successive QRT complexes. That is, QT should be less than 1/2 of the RR interval. P complex (P wave) is normally 0.04–0.11 s in duration. Its deviation from the normal wave shape or its disappearing means a pathological case. The normal duration of ST segment is 0.02–0.12 s. Any drop in the duration of the ST segment suggests ischemic, whilst its shift above the cycle-axis suggests a heart attack. The normal T-complex is about half of the P-complex time. When it is inverted (except for aVR lead), it typically indicates a heart attack. The aVR means the case of augmented unipolar lead [7], which is directed towards the RA electrode (−150°).

These characteristics of the ECG signal can be detected and compared with the standard signal pattern of the examined patient. In the case of a difference, the signal should be transmitted to the embedded system in the mobile phone. The possible shapes of the expected deviations can be determined by a physician and programmed to be a part of the holter complimentary embedded system.

**Figure 2.**
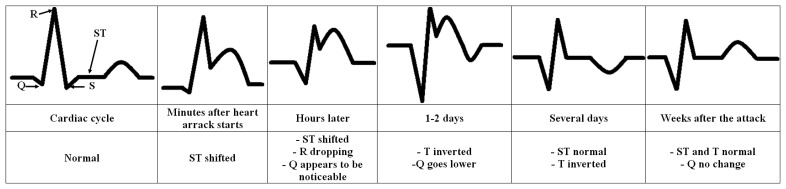
Recorded evolution of ECG waveform showing the pathological disturbances after the heart attack in ST, T and Q parts (basing on the knowledge acquired from [6]). Any pathological change in any stage can automatically be described as an abnormal disturbance.

Figure 2 shows the recorded evolution of ECG waveform with the pathological ST-segment and T-complex after the heart attack. It also shows the recorded changes in Q position. This is only an example of extracting disturbances from the ECG signal and hence checked on-line by computer.

The relatively short duration of QRS complex indicates that ventricular depolarization normally occurs very rapidly. If, for any reason, it is prolonged to have a value greater than 0.1 s, conduction is impaired within the ventricles. This means the ventricular foci (abnormal pacemaker site) becomes the pacemaker driving the ventricle. Such an ectopic foci nearly always results in impulses being conducted over slower pathways within the heart, thereby increasing the time for depolarization and the duration of QRS complex. This and any of the pathological changes in regular heartbeat pattern (Figure 1) and their potential cause to a noticeable disturbance in the heart work are easy to detect, record, analyze and hence to compare with the physiological state automatically. The under construction complimentary embedded system is planned to include all these tasks.

## 3. Related Work

Pervasive and ubiquitous computing systems and their applications in intelligent ambient environment are becoming more and more popular [8]. In [9] the idea of monitoring and transition of ECG signal of a heart-sick person stuck in a crowd is described. The signal is recorded at times already set by the patient and the detecting unit can be programmed to send a cardiac cycle to other mobile devices whenever needed. As mentioned above, the need comes when the ECG holter carrier requests help. The authors will show the nature of ECG waveform itself and then the expected cardiac cycle changes when disturbances in the heart rhythm are detected some reasonable time before heart failure takes place. Early diagnosed cardiovascular changes can potentially rescue the patient [10].

The concept of the described early warning mobile ECG analysis system originates from the authors' work described in [9], where the simulation of the reaction of surrounding crowd on the alerting message was conducted and the results were promising, although the general idea of such systems is as old as Personal Digital Assistants (PDAs), with some of more interesting approaches described in 2004 [11] using General Packet Radio Services (GPRS) transmission and in [12] using MMS for emergency station alerting.

The proposed system, partially described in [13], is divided into three parts:

- mobile phone/tablet software;
- home PC/laptop software;
- analytical software for doctors.

These three parts will work in a chain. First the software on a mobile device, connected in a simplest version to a 1-lead holter, will perform real-time analysis to detect any possible sudden life threatening situations, such as arrhythmia or myocardial infarction. This software will be responsible of alerting the nearest Emergency Station and people in close vicinity of the monitored patient in the aforementioned cases. The ECG data will also be stored and transferred (via cloud storage like in [14] or simply via Wi-Fi or a cable) to a computer to perform short- and long-term analysis to find minor and regular anomalies which may be early symptoms of future problems. In case of holters with more than one lead, monitoring of only one lead is necessary for detection of rapid changes, so the analysis of other leads will not be performed on mobile device. To lower resources consumption, the processing will be done on a personal computer (PC). The results of this analysis will be sent to a doctor for consultation and verification of a diagnosis. The authors' believe that no machine shall substitute experienced specialist.

**Figure 3.**
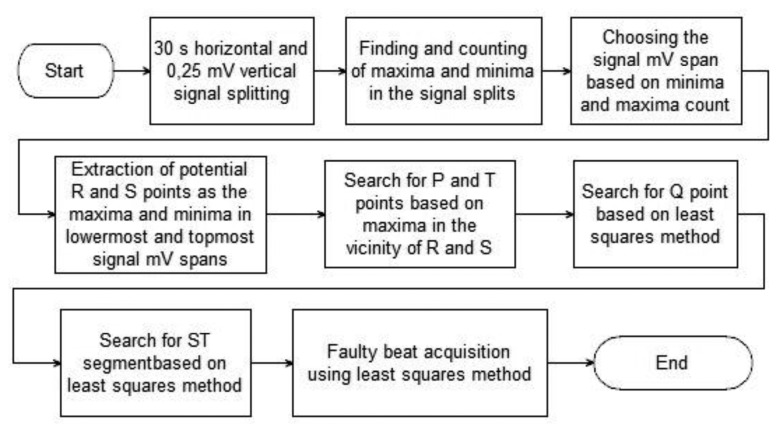
Flowchart of the heartbeats extraction algorithm.

The authors have recently developed almost all necessary algorithms for the system and are combining them into consistent, yet modular solution. Its main pieces, which were described in detail in an earlier publication of the authors [15], are the description of heartbeats and the acquisition of faulty beats. For the purpose of this paper the algorithms have undergone optimization and are used in a prototype implementation for mobile devices. The acquisition and description of proper heartbeats is conducted as described by the flowchart in Figure 3.

The algorithm presented in Figure 3 was developed by the authors and is based on statistical analysis of probabilities of the existence of characteristic points of ECG signal in the given millivolt (mV) and time frames. As mentioned earlier, the algorithm is described thoroughly in [15] so only a short description is presented here.

The signal is at first split into 0.25-mV high and 30-second wide spans and inside these spans all minima and maxima are counted. Basing on their numbers the span of the signal is estimated and the mV spans containing possible R and S points are acquired. On the basis of these spans the probable P and T points are found. The last part is the search for Q point and ST segment with the use of least squares approach. In this paper the solution is used as a training algorithm to obtain the ECG signal pattern using proper parts of signals, so offline anomaly detection and description part of the original algorithm is unnecessary.

The missing part of the current system in the authors' model is the wireless ECG signal recorder. There are two possibilities to solve this problem. The first one are commercial holters and the second are wireless electrodes. Such sensors for ECG signal registration are proposed for example in [16], where the authors use an AT-mega1281V microcontroller attached to electrodes to transmit the signal to a PC. The embedded microchip is necessary for initial preprocessing, especially denoising, as described in detail in [17]. A similar, even simpler sensor is proposed in [18].

Another issue with the mobile ECG analysis system is the placement and number of electrodes. For identification of life threatening situations 1-lead 2-electrode system is enough although for complex analysis 12-lead solution is recommended. Cao, Li, *et al.* addressed this issue in their work [19] by proposing novel placement positions of electrodes which are more convenient for patient and provide signals similar to a regular 12-lead combination.

With the advances in development of intelligent materials there are also other solutions to a problem with discomfort while wearing normal electrodes, which are ECG sensors embed into chairs [20] for monitoring of people performing desktop jobs and even in clothes [21].

Described above is only a very small part of current state of the art in ECG signal analysis. It is also proven that ECG signal might be a good biometrical identification solution [22], so it would not be difficult to incorporate this possibility into the authors' work and use it as a biometrical passport or even as a door key.

## 4. Method Description and Implementation for Mobile Devices

The authors are currently concentrating on development of the ECG analysis software for mobile devices and are at the prototype stage. With the reimplementation of most of the code from C++ to C# the backbone of the application is ready and will be encapsulated in additional functionalities which are described in the Discussion section.

### 4.1. Simulation

**Figure 4.**
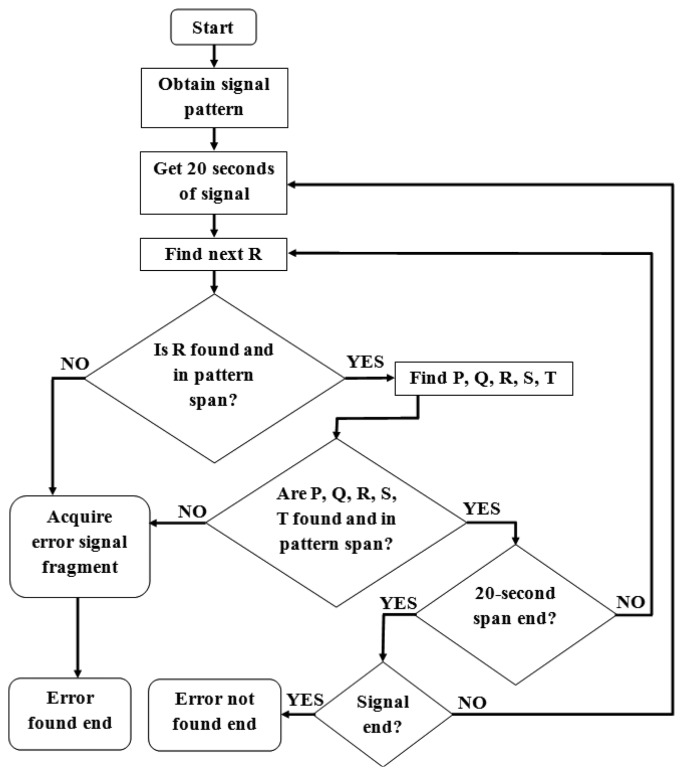
Flowchart of real time simulation.

The purpose of the simulation software is to create a simple analytical solution for the detection of anomalies consuming as little as possible resources. The flowchart of the simulation is presented in Figure 4. The software contains three main modules:

- signal description module, which is responsible for acquisition of proper signal characteristics,
- anomaly extraction and description module,
- real-time anomaly detection module.

The last module is in its prototype stage so the analysis stops when any anomalies appear for the first time. For the authors' current studies there is no necessity of further analysis of the nature of anomalies although this option is included in the simulation software and is under development for the mobile solution.

The first part, “Obtain signal pattern”, is performed as described in Figure 3. The analysis is performed on initial 1-2-minute-long fragments of the analyzed signals, although not longer than 20% of it. This way the average positions of characteristic points of the ECG of a patient are calculated. A pattern is obtained by defining both time and voltage interval for all characteristic points in conjunction with P point. The possible voltage difference from average values is defined as ±(maximum R − minimum S) × 0.001. Time difference interval is defined as ±0.5% of frequency.

**Figure 5.**
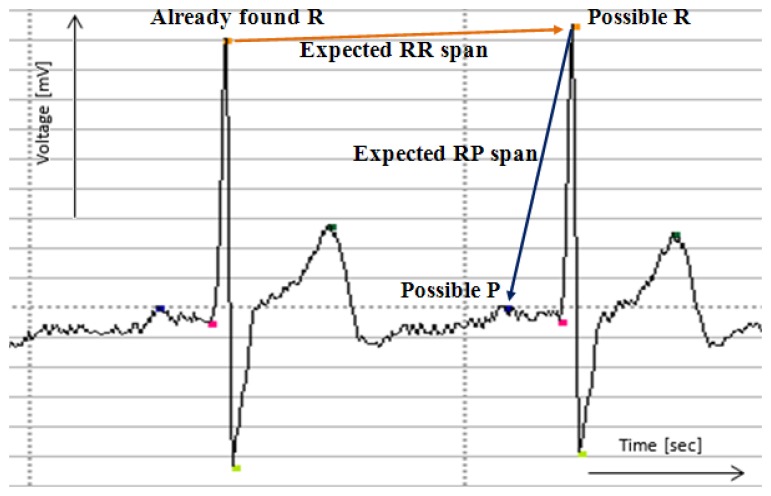
Finding of proper heartbeats on the example of R and P points. The signal and the markings of characteristic points are drawn by the authors' simulation software.

The rest of the signal is acquired and analyzed in 20-second-long spans (this time span is adjustable) which are attached to already sent earlier parts. Recently obtained 20-second-long span is widened for analysis with the end of the previous span, namely the part from the beginning of the last obtained proper heartbeat. This procedure ensures the continuity of analysis, which is based on earlier acquired patterns, as presented in Figure 5.

The next proper heartbeat is searched near the recently found one using average RR ratio. If the R point is found, the remaining characteristic points are found basing on expected distances from R point. The trend of ECG line between points is also checked using least square method to ensure proper shape of a signal. If no points are found in the expected interval or there are disturbances in the shape of a signal between points, the alert is raised.

If any of the characteristic points of the currently analyzed heartbeat are in close vicinity of interval border (depending on frequency, in the example it is 5% of the interval span) the interval is widened in that direction by |border − point value|.

This widening technique is sufficient for short signals which are used during the tests described in this paper. For longer signals the interval is adjusted every 5 to 15 min basing on the last 1–3 min of a signal. Current implementation includes manual setting of time parameters. The proposed solution allows the separation of receiving and analysis modules and the idle mode on multithreaded systems may be in future used for more thorough analysis of the acquired earlier signal.

### 4.2. Mobile Solution

**Figure 6.**
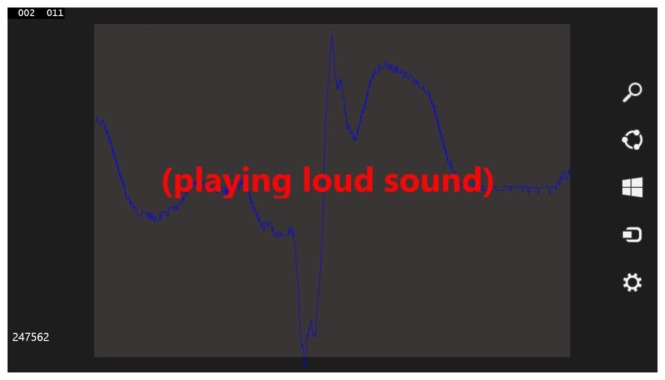
Tablet-dedicated software running on a virtual machine for presentation purposes.

The mobile solution is based on the described earlier simulation software and is very similar to it. Contrary to earlier software, it is created in C# and XAML using Visual Studio 2013 Ultimate and is easily portable between the Windows Phone 8 and Windows 8.1 Modern UI interfaces. There is no particular reason for this particular technology choice except the authors' preference, although similarities between C#/XAML Windows solution and JAVA/XML Android solution should significantly ease any future porting to more platforms. The software, containing an additional anomaly signaling module, is complete in terms of anomaly detection, however it is still in a prototype stage. It lacks the holter connection module and the Emergency Station notifying module. The lack of a holter connection means that the data is stored on and read from SD cards during tests, although 20-second-long signal acquisition (described in Section 4.1) is simulated. The mobile solution prototype is presented in Figure 6.

A virtual machine on regular PC is used for development and presentation purposes, namely for easier acquisition of screenshots and changing the code of the software on-the-fly. That is why debug information is also presented in Figure 6. The textbox with “(playing loud sound)” message was added to the software only for presentation purposes, typically in case of emergency there is no need of displaying any message on a patient's device as the loud sound is the preferable notification technique. The pseudocode of this solution is presented below:

1. Obtain the template of the signal for the specified person (as described in Figure 3)
2. Obtain the part of monitored signal (20 s)
3. Draw this part of the signal on a display
4. For each point in the acquired signal
  4.1. Find all points within R-value boundaries
  4.2. Group potential R points that are close to each other and find true potential R point among them
  4.3. Check RR spans with average RR span value for the person
  4.4. Analyze the neighborhood of R points to check whether it is possible to find P, Q, S and T points and whether they are in set boundaries
  4.5. Analyze the shape of the signal between found proper points to check its desired regularity:
    4.5.1. Apply the least square method to calculate *a* values for each point:
      double a;
      int *S_xx_* = 0, *S_x_* = 0, *S_y_* = 0, *S_xy_* = 0
      for each point (x;y) in neighbors
      *S_xx_* + = (*x* × *x*)
      *S_x_* + = *x*
      *S_y_* + = *y*
      *S_xy_* + = *x* × *y*
      *a* = (double)(*S* × *S_xy_* – *S_x_* × *S_y_*)/(double)(*S* × *S_xx_* – *S_x_* × *S_x_*)
  4.5.2. Determine whether the *a* values have ascending or descending pattern (depending on the analyzed point)
5. Adjust intervals of average heartbeat template of the person to current values
6. Go back to step 2. If *not the end of the signal* and *no anomalies are found*
7. If *anomalies are found*—raise alert
8. Analyze the anomaly to determine its type
9. Stop

As can be seen, this approach is very simplistic.

Step 5, as described in Section 4.1, is currently very simple. As mentioned before, the original description of the considered ECG signal bases on the characteristic points acquired from a short fragment of the signal. For each point the span *<minimum value of characteristic point—deviation; maximum value of characteristic point + deviation>* is defined. The new found points have to be contained in these set boundaries. When the found points are near the borders of the interval calculated for this point, the interval is expanded by fixed deviation. This solution is optimal for short signals, although for longer ones the better solution to step 5 is not obvious. This is because it has to handle the situations when a heartbeat frequency changes rapidly, for example when the person is surprised or frightened, but it also shall not be too flexible. The authors would determine proper spans and adjustability after experiments with longer signals.

Step 8 is currently under advanced development. The authors have completed the algorithm for acquisition of characteristic points from abnormal ECG fragments and their description (see [15]) although the software does not yet recognize the cause of the abnormality. It needs to be trained on abnormal samples to learn disease patterns. After acquisition of longer and more differentiated signals and extensive testing it will be added to the software.

## 5. Simulation Tests and Results

**Figure 7.**
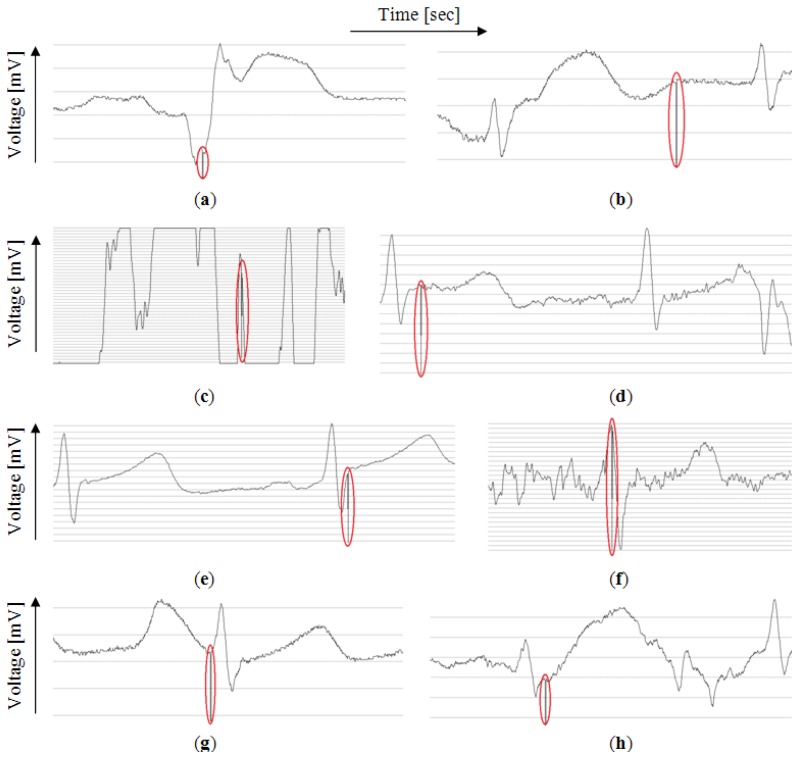
The anomalies that stopped the simulation. The signals are drawn by the authors' simulation software. (**a**) One faulty beat; (**b**) A series of faulty beats; (**c**) One of the electrodes probably fell off; (**d**) Anomalies in signal shape; (**e**) Higher than average T value; (**f**) Electrical distortion in signal; (**g**) Higher than average P value; (**h**) P and T waver merged.

The tests were performed on 50 signal samples with duration of few minutes of mothers' parts of the signals from the PhysioNet Non-Invasive Fetal Electrocardiogram Database [23]. The database contains 55 samples, five of which were removed because of technical problems with encoding. The samples are of various length of around 6 to 10 min. Of these samples eight contain anomalies and the rest is correct. Anomalies were detected properly in eight samples as presented in Figure 7.

The circles added to the figure mark the points in the signals where the algorithm started the analysis of the problem. In the test software, for better visual identification of such point, the value of the signal in this place was set to minimum possible value of the signal. The results were verified manually and proven to be correct.

It can be noticed that the procedure responds correctly to various types of anomalies although the point of stopping might be misleading because currently, as mentioned before, it is the point where the analysis of anomaly starts, not its actual beginning.

The extraction of faulty beats is now very simple. The whole part of the signal between the last correct heartbeat before the anomaly and the first correct heartbeat after the anomaly is extracted. True beginnings of the anomalies are not necessary considering the case of raising alert when the anomalies are detected, although in the simulation software they are found during anomaly description stage using linear least squares approach [15].

The same tests were also conducted on Asus Transformer Book T100 tablet using the mobile solution. The tests show that on average in 1 millisecond around 31,250 samples are analyzed which, with the signal frequency of 1000 Hz, gives on average around 30 s of signal analyzed every millisecond.

## 6. Conclusions and Discussion as a Basis of Future Work

Both the simulation and the mobile device tests proved that the proposed solution might be sufficient for the detection of rapid heartbeat shape and frequency changes. On the mobile device there is still enough time and computing power available for additional functionalities. This leaves a space for a possible optional module for deep analysis of ECG signals on mobile devices and sending already preprocessed anomaly descriptions to a doctor and an emergency station which will limit the amount of transferred data. During the tests only one of four cores of CPU of the device was used which also indicates that using the full computing power of the device may allow complete 12-lead ECG signal analysis in real time if such analysis were needed.

Having presented the authors' current work, let's discuss objectives and constraints of the proposed system. First of all the mobile system has to consume as little computing power as possible. This is due to the fact that no dedicated hardware will be used for processing and the device should also serve its original role with ease and not be limited with shortened operation time on battery. That is why the long- and short-term analysis shall be left to dedicated computers. The mobile software shall concentrate only on life threatening anomaly detection and raising alerts in case of an emergency. The authors are currently researching the best ways to notify a patient's surroundings, mainly who and how shall they be alerted (informing of nearest emergency station is unquestionable). The main questions are:

- Shall the notification of surroundings be only via a loud noise or should an extra alert be sent to the mobile devices of surrounding people?
- How to deal with the cases of people being alone—who should be alerted, shall it be family, neighbors, should the alert be sent to mobile phones in the range of the nearby mobile relay?

The authors consider context-based notifications, based on the place where the patient is and a list of contacts on their mobile device. For example, when the person is at home, the people to alert are either family (if they are at home) or neighbors (if the person is alone). If the person is at work, the colleagues in the same building shall be informed. The system could also be integrated with, for example, Google Maps (especially with the maps of the interiors of buildings) and draw a route to a person in trouble. The sound alert on both the patient's and the receivers' mobile devices shall also change depending on the distance between them to ease the finding of the patient. Global Positioning System (GPS) is used for this purpose, for example in [24], although GPS is not precise enough, especially inside buildings.

The second issue is the integration of mobile software with holters or sensors. The authors want to implement both USB, Bluetooth and Wi-Fi compatibility and are working on modular, easy exchangeable communication libraries to let the software be adjustable and communicate with both existing commercial holters and future wireless electrode solutions.

In general, all solutions proposed by the authors are being designed to be as modular as possible, with exchangeable algorithm packages and the possibility of easy addition of more medical features, such as for example blood oxidation, which also is in the spectrum of interests of authors [25], or blood pressure. There are also many more interesting possible functionalities of such system, like QR codes with instructions for medicine packs as being added to real life ECG monitoring system in [26].

The long term testing of the proposed algorithms and development of procedures which will adjust the description of reference signal (used in checking for anomalies) to daily routine and activities of monitored patient is also necessary and will be conducted during future research.
